# Interleukins and Ischemic Stroke

**DOI:** 10.3389/fimmu.2022.828447

**Published:** 2022-01-31

**Authors:** Hua Zhu, Siping Hu, Yuntao Li, Yao Sun, Xiaoxing Xiong, Xinyao Hu, Junjing Chen, Sheng Qiu

**Affiliations:** ^1^ Department of Neurosurgery, The Affiliated Huzhou Hospital, Zhejiang University School of Medicine (Huzhou Central Hospital), Huzhou, China; ^2^ Department of Neurosurgery, Renmin Hospital of Wuhan University, Wuhan, China; ^3^ Department of Anesthesiology, The Affiliated Huzhou Hospital, Zhejiang University School of Medicine (Huzhou Central Hospital), Huzhou, China; ^4^ Department of General Surgery, The Affiliated Huzhou Hospital, Zhejiang University School of Medicine (Huzhou Central Hospital), Huzhou, China

**Keywords:** interleukins, ischemic stroke, inflammation, cytokines, neuro-immune

## Abstract

Ischemic stroke after cerebral artery occlusion is one of the major causes of chronic disability worldwide. Interleukins (ILs) play a bidirectional role in ischemic stroke through information transmission, activation and regulation of immune cells, mediating the activation, multiplication and differentiation of T and B cells and in the inflammatory reaction. Crosstalk between different ILs in different immune cells also impact the outcome of ischemic stroke. This overview is aimed to roughly discuss the multiple roles of ILs after ischemic stroke. The roles of IL-1, IL-2, IL-4, IL-5, IL-6, IL-8, IL-9, IL-10, IL-12, IL-13, IL-15, IL-16, IL-17, IL-18, IL-19, IL-21, IL-22, IL-23, IL-32, IL-33, IL-34, IL-37, and IL-38 in ischemic stroke were discussed in this review.

## 1 Introduction

Ischemic stroke after cerebral artery occlusion is one of the major causes of chronic disability worldwide, and there is still a lack of effective methods to improve functional recovery after cerebral stroke (1). After ischemic stroke, a severe shortage of blood supply to the brain leads to the insufficient oxygen supply to the brain, which in turn leads to neuronal death. Inflammatory responses at the blood-endothelial interface of brain capillaries are the basis of ischemic tissue damage. Furthermore, inflammatory interactions at the blood-endothelial interface, including adhesion molecules, cytokines, chemokines and white blood cells, are crucial for the pathogenesis of tissue injury in cerebral infarction (2). Pathophysiological changes after ischemic stroke include ion imbalance, neuroinflammation, and abnormal activation of immune cells, can lead to neuronal death. However, despite extensive research work have been made, the exact mechanisms of stroke damage are not fully understood. It is clear that ILs play a major role in the progression of ischemic stroke disease.

IL, refers to a lymphocyte medium that interacts between white blood cells or immune cells. It is a cytokine in the same category as blood cell growth factor. Both IL and hemocyte growth factor belong to cytokines, and they coordinate and interact with each other to complete hematopoiesis and immune regulation functions together. IL plays a crucial role in information transmission, activation and regulation of immune cells, mediating the activation, multiplication and differentiation of T and B cells and in the inflammatory reaction (3). There is a close relationship between IL and the pathogenesis of ischemic stroke. This review is to discuss the inflammatory effects of IL in the pathogenesis of stroke, the interactions between different IL-mediated pathways, the cell-type dependent effects of different mediators and how different ILs regulate complex inflammatory cascades. The role of IL-1, IL-4, IL-6, and IL-10 were discussed in more detail.

## 2 IL-1 Family

IL-1, described earlier as a fever-causing protein called human leukocyte pyrogen, is one of the pro-inflammatory cytokines produced by monocytes, macrophages, and epithelial cells. The IL-1 family consists of IL-1α, IL-1β, and specific receptor antagonist (IL-1RN) (4). The IL-1 gene complex is located on chromosome-14 and consists of three linked genes, namely IL-1α, IL-1β and IL-β (5). IL-1α and IL-1β was regarded as pro-inflammatory cytokines. The sequence homology of IL-1α and IL-1β is not high, but they bind to the same receptor complex and have similar biological activity. IL-1RN is a 16-18 kD protein that binds competitively with IL-1 and its receptor to become an important anti-inflammatory cytokine. There are five alleles for IL-1RN, and IL-1RN*1 is the most common genotype, followed by IL-1RN*2. The incidence of the remaining alleles (IL-1RN*3, IL-1RN*4 and IL-1RN*5) is less than 1% (6). Among them, IL-1RN*2 polymorphism is considered to be a genetic risk factor for coronary artery disease and atherosclerosis, which is closely related to ischemic stroke.

### 2.1 Mechanism of Pleiotropic Effects of IL-1 on Ischemic Stroke

IL-1 is a multifactorial cytokine with multiple biological effects in many cell types, many of which are associated with stroke risk and outcome. Downstream effects of IL-1 include increased expression of cytokines, chemokines, and growth factors, activation of matrix metalloproteinases, upregulation of adhesion molecules, increased leukocyte infiltration, activation of platelets, alteration of blood flow, increased angiogenesis, decreased neurogenesis, and numerous other effects. We have discussed some of all these effects and related mechanisms in detail, and the rest can be found in the reviews (7, 8).

**Figure 1:**
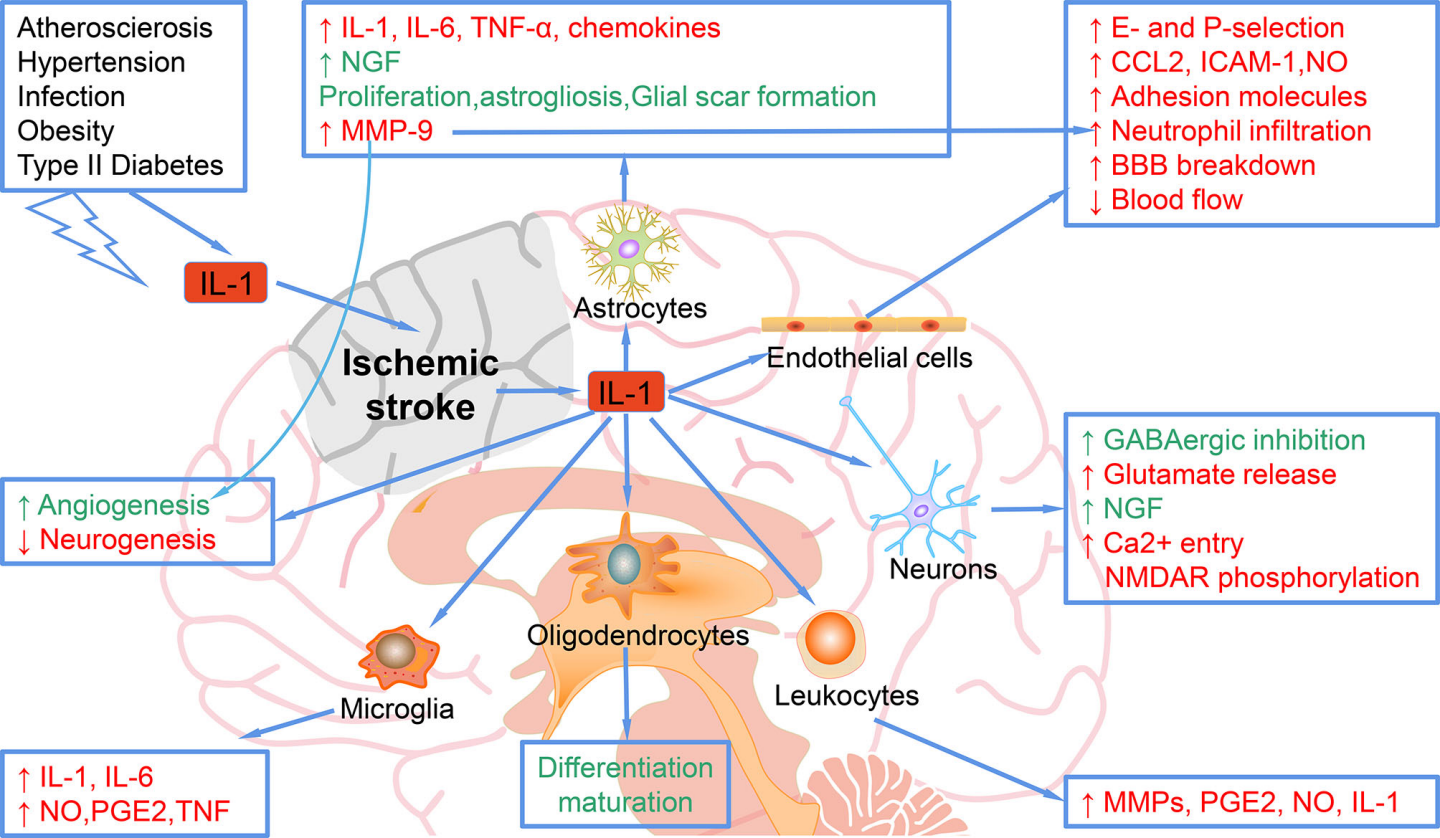
Mechanisms of the role of IL-1 in ischemic stroke. IL-1, elevated after ischemic stroke or other risk factors, can target astrocytes and up-regulate the expression of IL-1, IL-6, TNF-α, MMP-9, and chemokines. IL-1 target astrocytes to promote the NGF, proliferation, astrogliosis, and glial scar formation, which ameliorate ischemic injury. IL-1 targets endothelial cells and increases the E- and P-selection, CCL-2, ICAM-1, NO, adhesion molecules, neutrophil infiltration, blood flow decrease and BBB breakdown, which exacerbate ischemic stroke. When targets neurons, IL-1 increases the GABAergic inhibition and NGF, which rescue ischemic injury, while IL-1 also increases glutamate release and Ca2+ entry NMDAR phosphorylation, which cause more damage in ischemic stroke. Leukocytes can be regulated by IL-1 to secret more MMPs, PGE2, NO, and IL-1. In addition, microglia can be regulated by IL-1 to elevate the expression of IL-1, IL-6, NO, PGE2 and TNF. Oligodendrocytes can also be regulated by IL-1 for differentiation maturation.

Stroke-related comorbidities and risk factors are associated with elevated systemic inflammation, mediated in part by IL-1. As shown in **Figure 1**, in acute phase, the increase of IL-1 in the brain after stroke mediates the harmful the inflammatory process, including up-regulation of IL-6, TNF-α, MMP-9 and chemokines in astrocytes; inhibition of neurogenesis (9); increase of adhesion molecules and neutrophil infiltration, decrease of BBB integrity and blood flow by acting on endothelial cells, leading to worse outcomes. Moreover, IL-1 stimulates the proliferation and activation of astrocytes, leading to astrocyte hyperplasia, which is a typical response to brain injury. Data reported in many studies confirm that IL-1 upregulates a large number of genes in astrocytes, which encode neurotoxic factors including MMPs, chemokines, pro-inflammatory cytokines such as IL-6 and TNFα, but also survival-promoting mediators such as NGF (nerve growth factor) (10). IL-1 also exerts its effects on cerebrovascular endothelial cells to increase the production of chemoattractant and adhesion molecules such as CCL2 (CC chemokine ligand-2), ICAM-1 (intercellular adhesion molecule-1), and E- and P-selectin, and even promotes the breakdown of BBB, events that are associated with recruitment of leukocytes (11). IL-1 can act directly on neurons through an alternative signaling mechanism involving ceramide production and activation of Src kinase that phosphorylates the NMDAR [NMDA (N-methyl-D-aspartate) receptor] subunit 2B, leading to enhanced calcium entry and increased vulnerability to additional injury (12). IL-1 may also induce neurotoxicity indirectly through its action on the vascular endothelium to promote the recruitment of leukocytes, especially neutrophils that damage the neurovascular unit through the release of MMPs and reactive oxygen species (ROS) (11).

However, in the subacute and chronic phases post-stroke, some of the effects of IL-1 may be beneficial. For example, IL-1 promotes the glial scar formation and enhances angiogenesis, thereby promoting ischemic stroke recovery (13). In addition, IL-1 is not toxic to pure neurons in culture and can even promote survival through enhancement of synaptic GABA(γ-aminobutyric acid)ergic inhibition or production of NGF (14, 15).

### 2.2 The Mechanisms of IL-1β-Induced Brain Damage in Ischemic Stroke

In acute ischemic stroke, blood perfusion to the brain is reduced. In cerebral infarction, when blood flow is 10%-25% lower than normal, nerve cells will suffer irreversible damage or even death, and inflammatory cells in the tissue will release inflammatory factors. As one of the most powerful pro-inflammatory cytokines, IL-1β exerts an essential role in ischemic stroke mainly through the following mechanisms.

#### 2.2.1 IL-1β Aggravates the BBB Dysfunction

After ischemic stroke, increased secretion of IL-1β activates phospholipase A2 to degrade arachidonic acid and destroy the phospholipid bilayer (16). Moreover, the metabolites, prostaglandin and leukotriene, can promote the increase of microvascular permeability, resulting in blood-brain barrier (BBB) dysfunction and the formation of vasogenic brain edema (17). Meanwhile, after ischemic stroke, the reduction of glucose and oxygen supply, insufficient ATP production, and enhanced glycolysis, lead to the occurrence of cytotoxic brain edema. The interaction between the vasogenic brain edema and cytotoxic brain edema causes cranial pressure increase, secondary injury of brain tissue, and the possible occurrence of cerebral hernia in severe cases, endangering the life of the patient. In addition, IL-1β also aggravates ischemic injury by promoting the expression of adhesion molecules between endothelial cells, inducing leukocyte migration to the ischemic area to trigger inflammatory response (18).

#### 2.2.2 IL-1β Mediates the Inflammatory Response in Ischemic Stroke

IL-1β stimulates the activation of microglia, which, as the main effector cells in the neuroinflammatory response, aggravates the inflammatory response and leads to secondary brain damage by secreting and releasing a series of potential neurotoxic substances, such as TNF-α and iNOS. IL-1β activates IκB kinase through the IRAK pathway, resulting in the phosphorylation and ubiquitination of IL-1β-mediated IκB-α, which ultimately upregulates the expression of NF-kB in the cell nucleus and induces the increase of the transcription of target genes such as IL-8 and TNF-α (19). Study has demonstrated that IL-1β regulates the PI3K/AKT pathway to stimulate IL-6 and other cytokines, which synergistically act on ischemic areas and aggravate the damage effect (20). Other studies have shown that up-regulation of IL-6 and other pro-inflammatory cytokines can promote the phosphorylation of JAK2/STAT3 (21, 22). After P-STAT3 enters the nucleus, it will bind to the DNA sequence characteristic of the promoter region of target genes and up-regulate the transcription of IL-1β, IL-6 and TNF-α genes (22). This vicious cycle leads to persistent inflammation, and damaged brain cells fail to recover.

#### 2.2.3 IL-1β Promotes Apoptosis After Ischemic Stroke

After ischemic stroke, a large number of potentially salvageable neurons exist in the ischemic penumbra. However, with the prolongation of ischemia time, IL-1β promotes the apoptosis of the damaged cells by activating the apoptotic molecular mechanism, leading to the original ischemic penumbra gradually becoming the area of cerebral infarction, and finally the aggravated brain damage. Studies have verified that IL-1β plays a crucial role in the process of apoptosis of injured cells. This effect is mainly through the following two aspects: (1) activation of the excitatory toxicity mediated by glutamate (23); (2) activating the apoptotic cascade to activate the JNK/AP-1 pathway (24). Recently, it has been reported that the AIM2 inflammasome-derived IL-1β production activated triggers the expression of FasL in the spleen monocytes which evokes the apoptosis of Fas-dependent extrinsic T cells, causing an increased risk of infection by bacteria after ischemic stroke (25). Therefore, IL-1β may be involved in the signaling cascade activated by AIM2 inflammasome, causing immune suppression and secondary infection after stroke injury.

## 3 IL-2

Il-2, also known as cell growth factor, is an immunomodulatory lymphocyte secreted by T lymphocytes after being stimulated by antigen. In addition to maintaining the long-term multiplication and differentiation of T cells *in vitro*, IL also has important biological functions such as enhancing the killing activity of NK cells, promoting the proliferation and differentiation of B cells, and inducing the production of lymphokine-activated killer cells.

### 3.1 IL-2 Expression Decreased After Ischemic Stroke

Clinical trials showed that the level of serum IL-2 in patients with acute cerebral apoplexy was significantly lower than that in normal control group (26). The mechanism may be related to the following two factors: (1) In acute stroke, the body stress response, the immune stability *in vivo* is destroyed, especially the function of T cell is affected, so that the blood level of IL-2 is significantly decreased; (2) In acute stroke, the brain tissue cells are damaged, and local ischemia and hypoxia reduce the synthesis of IL-2 in the brain.

### 3.2 The Role of IL-2 in Ischemic Stroke

The IL-2/IL-2 antibody complex (IL-2/IL-2Ab) may improve the prognosis of ischemic stroke by regulating the amount of regulatory T cells (Tregs) in the body (27). Tregs are known to prevent ischemic stroke. However, the small amount of Tregs limits their clinical efficacy.

Previous research has showed that IL-2/IL-2Ab treatment selectively increases the amount of Tregs in the lymph nodes, spleen, and blood, significantly reduces the infarct volume, inhibits neuroinflammation, and improves sensorimotor function compared to stroke mice treated with isotype IgG (27). IL-2mAb has been reported to reduce demyelination after ischemic stroke by suppressing CD8 + T cells (28). The depletion of Tregs by diphtheria toxin eliminated neuroprotective effect provided by IL-2/IL-2Ab. IL-2/IL-2Ab promotes the expression of CD39 and CD73 in expanded Tregs, the deficiency of which may reduce the protective action of Tregs stimulated by IL-2/IL-2Ab in ischemic stroke mice (27). After stroke, increasing Treg cell numbers by delivering IL-2:IL-2 antibody complexes can improve white matter integrity and rescue neurological functions over the long term (29). In addition, Zhao et al. has found that ischemic stroke patients with poor functional outcomes at 3 months have significantly higher levels of IL-2 receptor α (sIL-2Rα) and lower levels of IL-2 than patients with good outcomes. Higher sIL-2Rα and IL-2 levels were associated with an increased and reduced risk of unfavorable outcomes, respectively (30), indicating that increased plasma sIL-2Rα and IL-2 levels manifested opposite correlations with functional outcome, illustrating the importance of IL-2/IL-2R autocrine loops in ischemic stroke.

## 4 IL-4

IL-4 regulates various immune responses, including the differentiation of T cells and nonspecific transformation of B cells (31). It is also the most characteristic M2 macrophage polarization promoter to date. Numerous evidences suggest that IL-4 plays a critical role in brain function under physiological and pathological conditions. For example, T-cell-derived IL-4 is essential for learning and memory in the normal brain. Levels of IL-4 in the brain tissue decrease with age, which may contribute to cognitive decline in older people and also increase the risk of Alzheimer’s disease (32). After ischemic stroke, IL-4 treatment has been shown to enhance white matter integrity (33).

### 4.1 IL-4 Promotes the M2 Polarization of Microglia/Macrophages

Recent animal and clinical researches have demonstrated the importance of IL-4 in the acute phase of stroke (34, 35). Several hours after the onset of stroke, the level of IL-4 in serum was observably increased (36). In addition, 24 hours after transient middle cerebral artery occlusion (MCAO), IL-4 deficiency resulted in brain injury and neurological dysfunction (37). IL-4 plays an important role in the M2 polarization and long-term recovery of microglia/macrophages after ischemic stroke. Mice lacking IL-4 have more M1-polarized microglia/macrophages, larger infarcts and more severe functional deficits after cerebral ischemia, while recombinant IL-4 can eliminate these effects (38). IL-4-polarized microglia cells may alleviate the ischemic stroke injury by promoting angiogenesis through the secretion of exosomes containing miRNA-26a (39). There is a direct salutary effect of IL-4 on oligodendrocyte differentiation that is mediated by the peroxisome proliferator-activated receptor gamma (PPARγ) axis. Additionally, PPARγ is essential for IL-4-induced oligodendrocyte progenitor cell differentiation and long-term functional improvements after stroke (33).

#### 4.1.1 Inhibition Pro-Inflammatory Cytokines

The neuroprotective effect of IL-4 may be achieved by stimulating IL-4/STAT6 signal transduction and inhibiting pro-inflammatory cytokines. Previous study has showed that IL-4 knockout mice produce more pro-inflammatory cytokines, including IL-1β and TNF-α (40). The loss of IL-4 in mice also increases sensitivity to mechanical pain.

#### 4.1.2 IL-4 Is Essential for Sex Differences in Vulnerability to Stroke

IL-4 protects against cerebral ischemia in male mice. However, female mice generally exhibit less damage in response to the same challenge of cerebral ischemia. Infarct volume in WT female mice in proestrus and estrus phases is markedly smaller than in males. IL-4 is required for female neuroprotection during the estrus phase of the estrus cycle (38). In protected female WT mice, microglia/macrophages were dominated by M2 polarization and inflammatory infiltration was reduced (40). Therefore, increasing macrophage M2 polarization, with or without added inhibition of inflammatory infiltration, may be a novel approach for stroke treatment.

#### 4.1.3 IL-4 Affects Neuronal Excitability

Chen et al. have shown that cortical pyramidal and stellate neurons common for ischemic penumbra after cerebral ischemia-reperfusion injury exhibit intrinsic hyperexcitability and enhanced excitatory synaptic transmissions in IL-4 knockout mice. In addition, upregulation of Nav1.1 channel, and downregulations of KCa3.1 channel and α6 subunit of GABAA receptors are observed in the cortical tissues and primary cortical neurons in IL-4 knockout mice (34), indicating that IL-4 deficiency results in neural hyperexcitability and aggravates cerebral ischemia-reperfusion injury.

## 5 IL-6

IL-6 is a glycoprotein with a molecular mass of 20 to 30 kDa, depending on the cellular source and preparation, and is a cytokine with pleiotropic, playing a role in central host defense (41). The IL-6 family of cytokines recruits gp130 for signaling. For IL-6 specifically, a hexamer forms (two IL-6, two IL-6R and two gp130) that can activate intracellular tyrosin-kinases such as JAK and, to a lesser extent, TYK, which, in turn, activate a number of proteins including the STAT family of transcription factors, or the RAS-RAF-MAPK pathway, PI3K, or IRS (insulin receptor substrate) (42). IL-6, mainly produced by monocyte macrophages, lymphoid cells, T cells, B cells, granulocytes, mast cells and endothelial cells, is a kind of multi-effector cytokine. IL-6 has critical effect in immune reactions, acute phase response and hematopoiesis regulation, mainly in autocrine or paracrine ways. By activating target genes, IL-6 not only serves as a differentiation and growth factor of hematopoietic cells, B cells, T cells, osteoclasts and endothelial cells, but also plays an important role in the growth, differentiation, regeneration and degradation of peripheral and central nervous system nerve cells. IL-6 activates and recruits neutrophils and monocytes, stimulates vascular endothelial cells to secrete adhesion molecules and other inflammatory transmitters, and enhances local inflammatory response (43). Circulating and local IL-6 production will lead to the state of pre-thrombosis, which can induce the production of platelet derived growth factor, fibroblast growth factor, TNF, macrophage colony stimulating factor, and promote the proliferation of smooth muscle cells (44).

### 5.1 Dual Role of IL-6 in Ischemic Stroke

The dysregulation of IL-6 is closely related to the occurrence and outcome of many clinical diseases, including coronary heart disease, leukemia, hypertension, ischemic stroke and so on (45). Su et al. demonstrated that elevated IL-6 induced by ischemia and hypoxia, oxidative stress, vascular occlusion and inflammation, partly leads to the production of the acute phase protein in the liver, thereby stimulating leukocyte recruitment and thrombosis, ultimately causing multiple cardio-cerebrovascular diseases including ischemic stroke (46). Elevated serum IL-6 levels are implicated in a higher risk of incident stroke and mediate the racial disparity in stroke *via* inflammatory effects of risk factors (47). Elevated plasma IL-6 has been reported to be a signatures of post-stroke delirium (48). Additionally, high IL-6 levels at 24 hours are associated with futile reperfusion in patients with acute ischemic stroke with large vessel occlusion treated with mechanical thrombectomy (49). Moreover, a lower admission level of IL-6 is positively correlated with the first-pass effect, which is defined as a complete or near-complete reperfusion achieved after a single thrombectomy pass is predictive of favorable outcome in acute ischemic stroke patients (50). These findings indicate that IL-6 may be a predictor of the prognosis of ischemic stroke patients.

**Figure 2:**
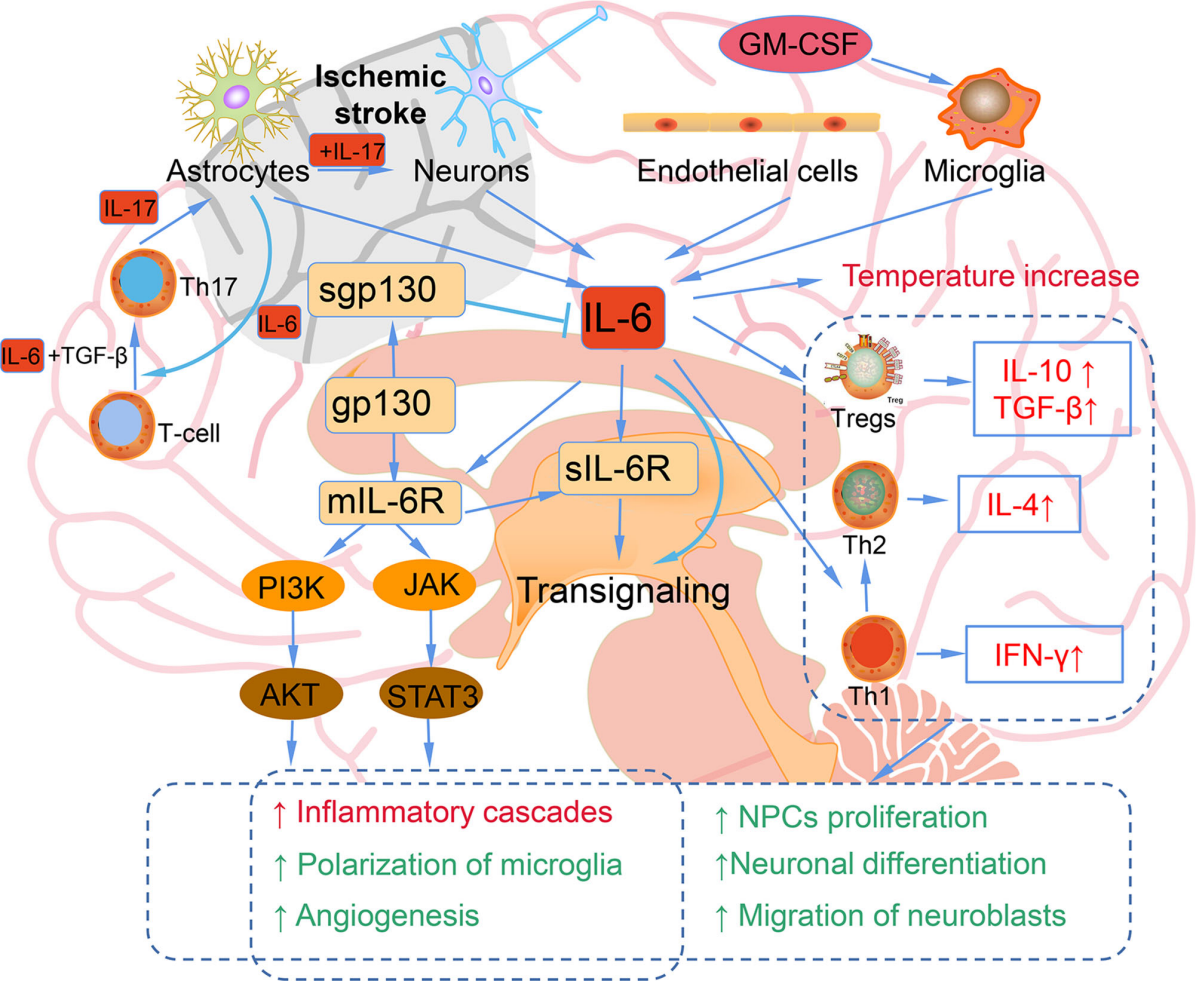
The bidirectional role of IL-6 in ischemic stroke. Astrocytes, neurons, endothelial cells, and microglia can produce IL-6 after ischemic stroke. Elevated IL-6 leads to the temperature increase; inflammatory cascades, microglia polarization, and angiogenesis mediated by JAK/STAT3 and PI3K/AKT pathways; increased IL-10, TGF-β induced by Tregs; Th1 polarizing to Th2; NPCs proliferation; neuronal differentiation; migration of neuroblasts.

IL-6 is a marker of inflammation after stroke, and elevated IL-6 is mainly secreted from neurons, microglia, astrocytes, and endothelia cells in the ischemic hemisphere, traditionally regarding as an adverse prognostic factor (51) (**Figure 2**). In the ischemic brain, IL-6 protein is mainly localized in the neurons of the cerebral cortex. The neuronal expression of IL-6 starts 3.5 h after ischemia, peaks after 24 h of reperfusion, and remains for 7 days. The immunoreactivity of IL-6 was most upregulated in ischemic penumbra. IL-6 released into the cerebrospinal fluid after stroke may lead to impaired cerebrovascular autoregulation and increased histopathology. In addition, IL-6 is related to the inflammation, which contributes to both damage and recovery process after ischemic stroke (52). The high levels of serum IL-6 have been reported to be related to the body temperature, early neurologic deterioration, infarct volume, and a long-term poor outcome. It has been identified that after stroke brain is the main source of IL-6 (53). In addition, inflammatory biomarkers, including C-reactive protein, fibrinogen, IL-1 receptor antagonist, and TNF-α are also elevated in parallel with IL-6 (54).

However, IL-6 is also a neurotrophic cytokines that shares a common receptor subunit, gp130, with other neurotrophic cytokines, such as leukemia inhibitory factor (LIF) and ciliary neurotrophic factor (55). The IL-6 expression is mainly observed in neuronal cells in the ischemic penumbra region, and the expression of LIF shows a similar pattern. The direct injection of these cytokines into the brain tissue after ischemic stroke can reduce cerebral ischemic damage. The main downstream signaling pathway of IL-6 is JAK-STAT, and the activation of STAT3 occurs primarily in neuronal cells after ischemic reperfusion. Since the role of STAT3 in stroke is also diverse and controversial, further studies are needed to explore the accurate action of STAT3 signaling in neuroprotective effect (54). IL-6 secreted from astrocytes promotes Th1 polarize into Th2 to mediate immunosuppressive microenvironment and contribute to the neurogenesis and angiogenesis and neuronal differentiation (51). IL-6 stimulates the phosphorylation of STAT3 and the early transcriptional activation of angiogenesis-related genes, thereby leading to the enhanced angiogenesis and elevated cerebral blood flow in the delayed period after ischemic stroke. IL-6R simultaneously activates the PI3K/AKT and JAK-STAT pathways, which play vital roles in angiogenesis after ischemic stroke (56). Additionally, IL-6 also been reported to facilitate post-traumatic healing in the CNS through repair of endothelial cells, which also demonstrates that IL-6 may enhance revascularization or angiogenesis after ischemic stroke (57). IL-6 increases CNS neuronal survival and decreases excitotoxic neuronal damage against NMDA-mediated injury and protects neurons against apoptosis (54). Continuously injection of recombinant for 7 days IL-6 into the lateral ventricle of gerbils subjected to transient cerebral ischemia, IL-6 injection was found to prevent learning disabilities and delayed neuronal loss (58). In conclusion, IL-6 has a dual effect in ischemic stroke, acting as an inflammatory factor in the acute stage and a neurotrophic mediator in the subacute and prolonged phase.

## 6 IL-8 in Ischemic Stroke

IL-8 is a chemotactic cytokine that promotes the chemotaxis of inflammatory cells and induces cell proliferation. After ischemic stroke, IL-8 levels are increased (59, 60), mobilizes and activates neutrophils, causing neutrophils to infiltrate into the ischemic area, aggravating the local inflammatory response, leading to the expansion of ischemic lesions, and leading to severe morbidity and disability (2, 61–63). Endothelin-1 may be a stimulator of IL-8 (62). IL-8 may be also involved in recruiting blood polymorphonuclear leukocytes to the sites of cerebral ischemia (59). The expression of pro-inflammatory cytokines (IL-1β, IL-6, IL-8, TNF-α) in the cortex of ischemic stroke mice was detected after the occurrence of cerebral ischemia (64). One study showed that the levels of these pro-inflammatory cytokines in patients with acute cerebral ischemia were observably higher than those in the control normal group, and the degree of disability in early phase of acute stroke was positively correlated with the level of serum IL-8 (65). IL-8 gene knockout has been shown to promote neuroglial cells activation while inhibit neuroinflammation through the PI3K/Akt/NF-κB-signaling pathway in mice with ischemic stroke (66). The high serum IL-8 levels are associated with prognosis. IL-8 exaggerates the ischemic stroke injury through inducing neutrophil-mediated-inflammation (61). The development of new neuroprotective treatments aimed to prevent neutrophil-mediated-inflammation induced by IL-8 is critical in the treatment of stroke, and prevention of clinical worsening. IL-8 can be used as important indicator to judge the severity of the early condition of acute ischemic stroke patients (67). However, IL-8 stimulates VEGF production in human bone marrow mesenchymal stem cells partially *via* the PI3K/Akt and MAPK/ERK signal transduction pathways and that administration of IL-8-treated human bone marrow mesenchymal stem cells increases angiogenesis after stroke (68).

## 7 IL-10

IL-10 is a significant anti-inflammatory cytokine that has inhibitory effects on a variety of immune cells. IL-10 was first identified in mouse Th2 cells, and was subsequently found to be secreted in astrocytes, neurons, B cells, monocytes/macrophages, keratinocytes, and human Th1 cells. IL-10 has strong anti-inflammatory and immunosuppressive activity. It can inhibit the production and release of IL-2, IFN-γ and pro-inflammatory factors, reduce the expression of immune receptors, inhibit human Th2 cells, lead to cell proliferation, cytokine production reduction (69). IL-10 binds to IL-10 receptors (IL-10R) to decrease inflammation and limit apoptosis (70). These effects make it a very important role in the protection of cerebral ischemia.

### 7.1 IL-10 in Ischemic Stroke

The neuroprotection of IL-10 on ischemic stroke has always been a research hotspot. A meta-analysis exploring the relationship between IL-10 gene polymorphism and ischemic stroke risk revealed no overall significant association of IL-10 with ischemic stroke risk, but an association was found with macrovascular disease and microvascular disease (71), demonstrating that certain subtypes of ischemic stroke are correlated to IL-10 gene polymorphisms.

In experimental stroke, the levels of IL-10 mRNA and protein and IL-10R mRNA were elevated, with IL-10 observed in microglia and IL-10R on astrocytes in the ischemic penumbra (72). In transgenic mice that overexpressed IL-10, infarct size was reduced, and apoptosis was limited 4 days post ischemic stroke (73). Additionally, Overexpression of IL-10 enhances the neuroprotective effect of mesenchymal stem cell transplantation through anti-inflammatory regulation, thus supporting the survival of neurons during acute ischemia (74). Both systemic intravenous (IV) and central intracerebroventricular (ICV) exogenous administration of IL-10 reduced infarct size after permanent MCAO (pMCAO) (75). Moreover, low IL-10 levels were related to poor stroke outcomes and a delayed, exacerbated inflammatory response that was alleviated by IL-10 administration after pMCAO (76). Lower levels of IL-10 and IL-33 may also be used to predict post stroke depression (77, 78). The IL-10 expression in the brain tissue increases with pathological changes of the central nervous system, promotes the survival of gliocyte and neurons, and inhibits inflammatory responses through multiple signaling pathways. Previous research showed that the significant decrease of IL-10 was significantly associated with the degree of neurological impairment, and the concentration of IL-10 had a high predictive value on the early neurobehavioral performance post-acute stroke (79). However, stroke patients are susceptible to infection as a result of stroke-induced immunosuppression, and elevated serum IL-10 levels have been identified as an independent predictor of post-stroke infection (80, 81). IL-10 overreaction can lead to immunosuppression and worsening neurological prognosis after stroke, indicating that IL-10 therapy should be used with caution (82). Elevated IL-10 levels may be associated with higher incidence of post-stroke urinary tract infection, leading to poorer recovery after ischemic stroke in women (83, 84). In addition, IL-10 can mediate the function of Th2 cells, exert a protective effect, and lead to the reduction of ischemic infarction lesions (85). Future studies should be aimed at differentiating between central and peripheral IL-10 effects post-stroke.

#### 7.1.1 The Mechanism of IL-10 in Inhibiting Inflammatory Responses After Stroke

**Figure 3:**
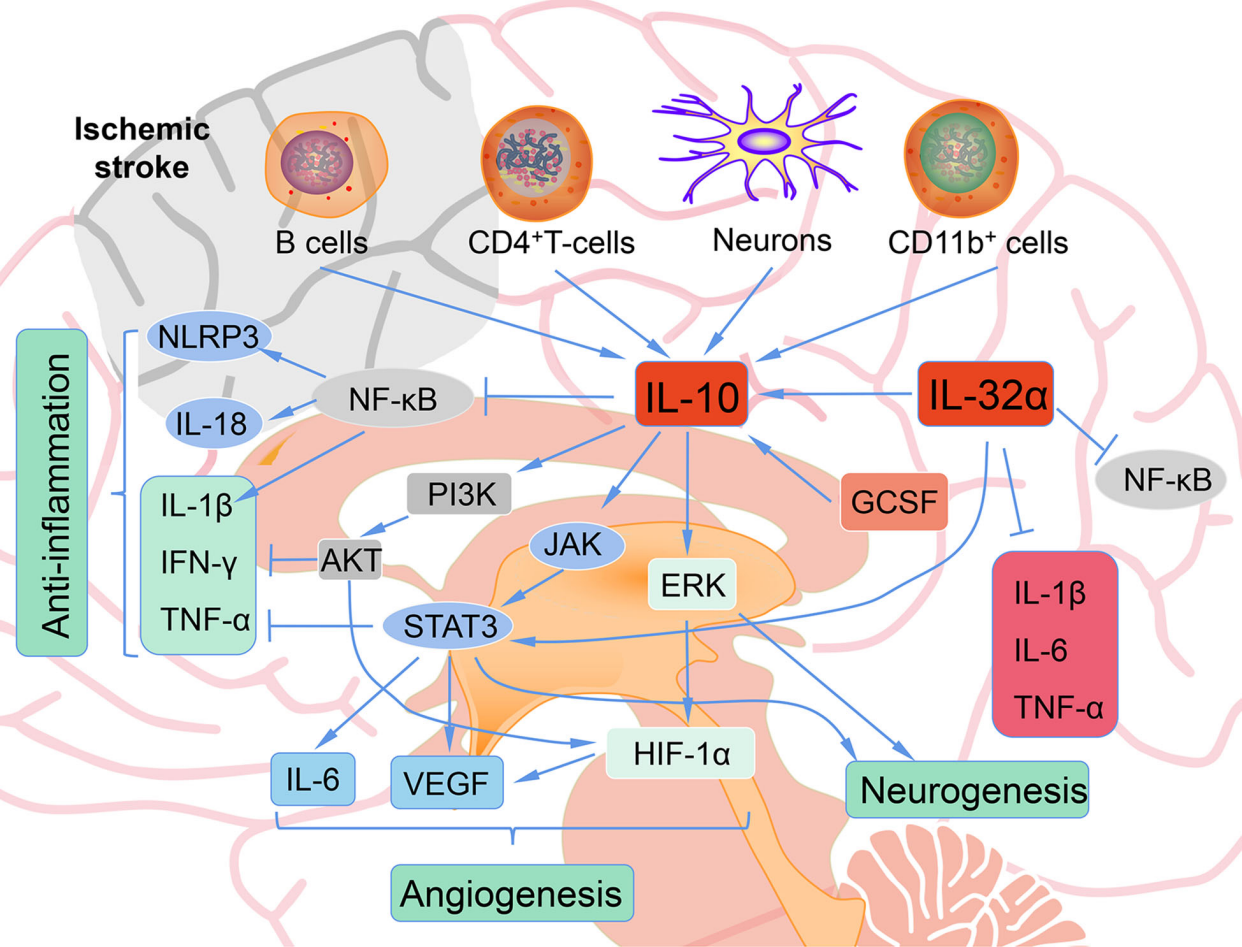
The role of IL-10 after ischemic stroke. B cells, CD4+ T cells, neurons, CD11b+ cells produce IL-10 after ischemic. IL-10 shows anti-inflammatory, angiogenesis, and neurogenesis role after ischemic stroke.

Immune cells, including T and B cells, are important in ameliorating neuroinflammation *via* the modulation of varieties of cytokines and chemokines, with IL-10 playing a central immunomodulatory role (86, 87). The protective effect of IL-10 on stroke is mainly achieved by inhibiting inflammatory reactions. Firstly, IL-10 decreased the expression and activity of pro-inflammatory cytokines such as IFN-γ, IL-1β and TNF-α through PI3K and STAT3 activation (**Figure 3**) (88). Secondly, IL-10 inhibits the synthesis and activity of Th1 lymphocytes (89). In addition, IL-10 treatment can effectively down-regulate the up-regulated pro-inflammatory signals in acute ischemic lesions after stroke, and can provide neuroprotection for ischemic stroke (90). IL-10 gene transduction before cerebral artery ischemia can alleviate brain damage induced by ischemia/reperfusion in rats through increasing the expression of heme oxygenase (91). IL-10 also exert its anti-inflammatory effects partially through inhibition of NF-κB (92). Hydrogen sulfide donor administration during reperfusion protects the integrity of BBB after ischemia/reperfusion and is accompanied by increased IL-10 expression, reduced NF-κB nuclear translocation, and MMP-9 and NOX4 activity (93). In MCAO mice, by reducing the release of neuroinflammatory factors (IL-6, IL-1β, TNF-α) and astrocyte activation, IL-32α overexpressing transgenic mice showed reduced cell death of ischemic neurons and enhanced anti-neuroinflammatory factor (IL-10), indicating a crosstalk between IL-32α and IL-6, IL-1β, IL-10 (94). IL-10-secreting CD4^+^ T cells induced by nasal MOG reduce injury following stroke. IL-10 secreted from CD4^+^ T cells may be the reason of the neuroprotection of oligodendrocyte glycoprotein administration in MCAO mice (95, 96). Increased IL-10 levels also decreased the number of CD11b^+^ cells that may contribute to secondary infarct expansion *via* nitric oxide pathways (96). Expansion of the CNS Treg cell population by administration of the CD28 superagonist monoclonal antibody at the start of reperfusion decreased the infarct volume 7 days after MCAO, and its effect was attributed to the increased IL-10 (97). Transfer of IL-10-producing B cells into B cell-deficient mice 24 h after MCAO attenuated cerebral ischemia-reperfusion injury, reduced the amount of T cells and monocytes in cerebral parenchyma, and improved the peripheral proinflammatory homeostasis (87). Interesting, IL-10-producing B cells also upregulated the number of Tregs (87), suggesting that there may be a positive feedback loop between B cells and Tregs, both of which play a neuroprotective role through IL-10 production. These facts indicate a complicated network between IL-10 and immune cells in ischemic stroke. These methods of targeting IL-10 to prevent recurrence of stroke may be realized in the interventional treatment of stroke.

#### 7.1.2 The Role of IL-10 in Neurogenesis After Ischemic Stroke

Injection of activated Tregs into the lateral ventricle of C57BL/6 mice 60 min after of transient ischemia promotes the proliferation of neural stem cell in the subventricular region in ischemic brain tissues. Moreover, this effect was abolished by blocking IL-10 with a neutralizing antibody, suggesting that activated Tregs act through IL-10 to facilitate the proliferation of neural stem cells (98). Hematopoietic cytokines such as GCSF and stem cell factor have been confirmed to promote neurogenesis (99), and also may be required to provide the initial signals for IL-10 production in ischemic stroke (100). Administration of these cytokines early (1–10 days) and later (11–20 days) post MCAO significantly elevated the mRNA expression of IL-10, reduced the activation of microglia/macrophages, and did not change proinflammatory cytokine levels in C57BL/6J mice (100). A study where bone marrow-derived mesenchymal stem cells were transplanted into the lateral ventricle of Sprague-Dawley rats before pMCAO yielded similar results, where IL-10 mRNA and protein levels were elevated 4 days post-stroke, TNFα was reduced, infarct size was smaller, and neurologic function was improved (101). IL-10 targets Nestin^+^ progenitors and activates neurogenesis by modulating ERK and STAT3 activity in adult subventricular zone (102). Either administration of stem cells themselves or hematopoietic cytokines may ameliorate ischemic stroke injury partially through the increase of IL-10. Additionally, neuroprotection after dysbiosis depends on IL-10 and IL-17. IL-10 is required for Treg mediated IL-17^+^ γδ T suppression (103). Recently, it has been reported that IL-10 acts differentially on αβ and γδ T cells. IL-17A producing CD4+ αβ T cells are directly controlled *via* their IL-10-receptor (IL-10R), whereas IL-10 by itself has no direct effect on the IL-17A production in γδ T cells. The control of the IL-17A production in γδ T cells depended on an intact IL-10R signaling in Tregs (104).

## 8 IL-12 and ischemic stroke

During antigen presentation to naive T cells, IL-12, IL-23, and IL-27 are produced by activated antigen-presenting cells, while IL-35 is a product of B cells and Tregs (105). The primary target cells of IL-12 are NK and T cells, which are stimulated to produce cytokines, proliferative and cytotoxic activities (106). IL-12 is produced early in infection and plays a pro-inflammatory role in the immune response, and serve as a cofactor in the polarization of T cell response to cell-mediated immunity (107).

Previous studies have confirmed that IL-12 plays an essential role in the pathological process of acute ischemic stroke (108). Cytokines are usually released in response to tissue injury, so increase levels of IL-12 in serum in patients with acute cerebral infarction are consistent with a rapid increase IL-12 levels in the serum of patients with acute myocardial infarction and severe brain injury (109). The increase of IL-12 in serum of stroke patients appears to be a local or systemic immune response to ischemic brain injury, and the increase of IL-12 in serum may be caused by the release of cytokines from the infarcted brain region or cerebrospinal fluid to the periphery. On the other hand, stroke-activated cerebral endothelial cells secrete cytokines that may activate peripheral blood monocytes, leading to systemic expression of cytokines (110). An increase in the number of IL-12 secreting monocytes and monocytes isolated from peripheral blood of patients with cerebral ischemia has been demonstrated (111, 112). In this case, the increase in serum IL-12 levels in patients with stroke may be caused by cytokines.

IL-12 may promote the deterioration of ischemic brain injury *via* cytokines by activating the ability of immunoreactive cells and modulating their abilities in response to inflammation. IL-12 increases the production and action of several pro-inflammatory cytokines and chemokines and promotes endothelial cells to release adhesion molecules, which are potent chemical attractors for different subsets of white blood cells, including monocytes and neutrophils (113, 114). Additionally, in a mouse cancer model, IL-12 gene therapy is associated with increased tissue infiltration and apoptosis of NK and T cells, which are important mechanisms of neuronal death induced by aggressive inflammatory cells in the ischemic brain (115). At present, we can know exactly that there is close correlation between increased levels of IL-12 in the ischemic stroke patients and reaction intensity in acute phase, the size of the early brain injury, neurological stroke severity and functional disability, suggesting IL-12 may play a critical role in pathophysiology of cerebral ischemia.

## 9 IL-13 and Ischemic Stroke

IL-13 is a protein secreted by activated T cells and is a powerful regulator of human monocyte and B cell function *in vitro* (116). IL-13 shares a common biological activity with IL-4 (117). IL-13 can induce the differentiation of mononuclear cell, inhibit LPS-induced mononuclear factor secretion, control inflammatory response, induce the proliferation of B cell and synthesize IgE antibody, cooperate with IL-2 to stimulate NK cells to produce IFN, thereby promoting mononuclear macrophage activation.

Previous studies have confirmed the indispensable role of IL-13 in ischemic stroke. IL-13 exerts an effect on microglia and infiltrating macrophages in the brain after stroke, and it can regulate the spontaneous polarization transition from anti-inflammatory to pro-inflammatory phenotype of microglia and macrophages (118). As a well-known modulator of immune response *in vitro*, IL-13 has been shown to have neuro-protective abilities in several experimental models of neurodegenerative diseases by significantly reducing the secretion of pro-inflammatory factors, reducing inflammatory cell infiltration, and inhibiting axonal loss as well as inducing anti-inflammatory microglial/macrophage responses (119, 120). Interestingly, interleukin-13 can also improve ischemic liver gluconeogenesis and hyperglycemia in stroke model rats (121), exerting a salutary action. That is, it has been demonstrated that mesenchymal stem cells that continuously secrete IL 13 can differentiate microglia and macrophages into neuroprotective M2 phenotypes in the pro-inflammatory state of ischemic stroke (122).

## 10 IL-15 and Ischemic Stroke

IL-15 can be produced by activated mononuclear macrophages, epidermal cells and fibroblasts. Its molecular structure has many similarities with that of IL-2, and it plays a similar biological activity to IL-2 (123). IL-15 also has the ability to chemotaxis and promote survival, and it can be involved in neuroinflammation. IL-15 also acts as an effective chemotactic agent for T cells, promoting the migration of T cells to inflammatory tissues (124). In addition, IL-15 maintains homeostasis and cytotoxic activity in lymphocytes (NK and CD8^+^ T cells) carrying its receptor (125).

Although recent studies have shown that astrocytes are a major source of IL-15 in the inflammatory central nervous system (126, 127), the potential role of IL-15 in astrocytes in cerebral ischemic injury is not completely clear. However, significant increase in IL-15 expression in astrocytes post ischemia reperfusion has been observed. Subsequent studies have shown that IL-15 is a key factor for astrocytes to control the degree of central nervous system inflammation and brain injury following ischemic stroke (126). Astrocytes produce inflammatory cytokines such as IL-15, which promote the cell-mediated immune reaction to ischemic stroke, increase the number of CD8^+^ T cells and NK cells, participating in ischemic nerve injury. In addition, blockage of IL-15 decreased the effector capacity of NK, CD8^+^ T and CD4^+^ T cells in WT mice after CIRI, and the elimination of IL-15 response after CIRI improved brain damage in adult mice (127). Moreover, IL-15 as a mediator of the crosstalk between astrocytes and microglia that exacerbates brain injury after intracerebral hemorrhage (128). Recently, IL-15 has been reported to modulates the response of cortical neurons to ischemia through alleviating endoplasmic reticulum stress and increasing cell survival (129). Therefore, therapy targeted IL-15 is a potential strategy for cerebral ischemia.

## 11 IL-16 in Ischemic Stroke

IL-16 is a pro-inflammatory cytokine produced by activated CD8^+^ T cells and activates CD4^+^ T cells, monocytes, macrophages, and dendritic cells by binding to the CD4 molecule (130). In addition, IL-16 promotes the production of inflammatory cytokines such as TNF-α, IL-1β, and IL-6, which has key effects in immune responses after ischemic stroke (131). Although the mechanism by which IL-16 acts as a mediator of inflammation is not fully understood, previous study has shown that IL-16 is involved in inflammatory disease through the activation of T lymphocytes and the expression of inflammatory cytokines (132). In the early stage of cerebral ischemia, T lymphocytes are activated to release reactive oxygen species, which eventually lead to brain damage. In later stages, T lymphocytes regulate brain recovery and regeneration. The depletion of T cells in the acute phase of ischemia reduces the infarction size and has a sustained protective action against ischemic stroke in the later stages of infarction development (133). IL-16 accumulates during injury-related response areas and perivascular areas through the infiltrated immune cells (e.g., neutrophils, CD8^+^ lymphocytes, and activated CD68^+^ microglia/macrophages) (134). The recruitment and activation of immune cells lead to microvascular aggregation and disorder of BBB, leading to secondary injury.

## 12 IL-18 in Ischemic Stroke

IL-18 is known as a pro-inflammatory cytokine. IL-18 expression is mainly observed in neuronal cells at early phase and in microglia at a later stage. IL-18 is associated with stroke-induced inflammation and that initial serum IL-18 levels may be predictive of stroke outcome. IL-18 KO mice exhibit the resistance to spatial restraint stress and CIRI (135). Caspase-1 activated by NLRP3 inflammasome, cleavage pro-IL-1β and pro-IL-18 to mature forms (IL-1β and IL-18), and mediate the inflammatory response or initiate the process of inflammatory cell death and pyrolysis. In addition, increased IL-18 in the brain causes depression-like behaviors by promoting the IL-18 receptor/NKCC1 (a sodium-potassium chloride co-transporter) signaling pathway. Hence, agents that inhibits NLRP3 inflammasome exert a neuroprotective effect on ischemic stroke and post-stroke depression *via* suppressing the expression of IL-18.

## 13 IL-19 and Ischemic Stroke

IL-19 is a member of the IL-10 family, which includes IL-10, IL-19, IL-20, IL-22, IL-24, and IL-26 (136). IL-19 was first discovered in primary human monocytes stimulated by LPS and GM-CSF (137). Subsequent reports on IL-19 mainly focused on its role as a product of immune cells. In immune cells, IL-19 is mainly secreted by monocytes, and a small part is expressed by B cells. It has been reported that IL-19 treatment can mature human T cell polarize them from pro-inflammatory Th1 phenotype to anti-inflammatory Th2 phenotype (138, 139). In addition, the anti-inflammatory effect of IL-19 on vascular diseases has also been clearly demonstrated (140).

As an anti-inflammatory factor, IL-19 also exerts a critical action in the immune reaction after the onset of ischemic stroke. Studies have shown that IL-19 can reduce infarct size and reduce neurological impairment after ischemic stroke through its anti-inflammatory ability (141). Moreover, IL-19 treatment can significantly reduce the up-regulation of TNF-α and IL-6 mRNA expression after ischemic stroke, inhibit the increase of microglia, macrophages, CD4^+^ T cells, CD8^+^ T cells, and B cells, and suppress the activation of macrophages and neutrophils. The administration of IL-19 also contributes to preserve the reduced number of immune cells, including macrophages, CD4^+^ T cells, CD8^+^ T cells, and B cells in peripheral blood compared to controls. In conclusion, IL-19 reduces cerebral infarction and neurologic deficits after cerebral ischemia in mice, possibly by inhibiting the infiltration and activation of immune cells and the increased expression of pro-inflammatory cytokine genes. Therefore, IL-19 may be identified as a new therapeutic agent to suppress the development of neuroinflammation after ischemic stroke.

## 14 IL-20 in Ischemic Stroke

IL-20 is also a member of the IL-10 family, and is produced by monocytes, epithelial cells, and endothelial cells. IL-20 has been related to a variety of inflammatory diseases, such as psoriasis, rheumatoid arthritis, atherosclerosis, and renal failure (142). IL-20 also induces the production of IL-6 (143), which is also a major pro-inflammatory cytokine. In addition, the levels of IL-6 in serum are correlated with cerebral infarction volume and stroke severity. IL-20 may be associated with the increased IL-6 levels in serum after ischemic stroke.

### 14.1 IL-20 Promotes Inflammation After Ischemic Stroke

The pathogenicity of IL-20 in ischemic brain injury has been demonstrated in transient MCAO animal models. After cerebral ischemia reperfusion, the levels of IL-20 in serum and ischemic penumbra were significantly elevated than sham groups, and glial cells were the main source of IL-20. After cerebral ischemia, hypoxia also induces the production of IL-20 in endothelial cells (144). The upregulation of IL-20 on glial pro-inflammatory cytokines and chemokines (may cooperate with IL-1β to promote inflammatory activity) is associated with inflammatory response and brain damage after ischemic stroke (145). Inflammatory cytokines and chemokines such as IL-1β, IL-8 and monocyte chemotactic protein 1 (MCP-1) are involved in the inflammatory response of infarcts (146). In conclusion, IL-20 is a novel hypoxia response factor that is upregulated in gliocyte after experimental ischemic stroke and mediates cell proliferation, signal transduction, and cytokine production. These suggest that IL-20 is related to the pathogenesis of cerebral ischemia, and IL-20 antagonists may have clinical therapeutic effects on ischemic stroke.

## 15 IL-23/IL-17

The IL-23/IL-17 axis has essential effect on the development of chronic inflammation and host defense against bacterial infection (147). In chronic inflammation, antigen-stimulated dendritic cells and macrophages produce IL-23, promoting the development of Th17 cells (148). Th17 cells produce IL-17, which promotes T cell activation by inducing the expression of various of inflammatory cytokines and triggers a powerful inflammatory response. IL-23 also acts on dendritic cells and macrophages in an autocrine/paracrine manner to promote the production of inflammatory cytokines, such as IL-1, IL-6, and TNF-α (149).

### 15.1 IL-23/IL-17 in Ischemic Stroke

Studies have shown that routine CD172a^+^/IRF4^+^ 2 type dendritic cells (CDC2s) are the main source of IL-23 in the brain following ischemic stroke, and are essential for IL-17 expression in γδT cells (148). Dendritic cells infiltrate the peri-infarcted area near the blood vessels after stroke. These cells induce γδT cells to produce IL-17, promoting neutrophil recruitment to the ischemic hemisphere (150). However, IL-23R gene knockout has no significant effect on the mortality in mice, suggesting that DC cells exert their adverse effects not only through IL-23, but also through other mechanisms (148). Additionally, IL-23 and IL-17 have been reported to increase after stroke, but there is insufficient clinical discriminatory power to predict the outcome of stroke (151). Vγ4 T cell-derived IL-17A, and IL-1β/IL-23 in infract hemisphere coordinately to exaggerate the inflammatory cascades and exacerbate ischemic tissue injury (152).

## 16 IL-33 and ischemic stroke

As a member of the IL-1 family, IL-33 can bind to membrane receptors on target cells to mediate downstream signaling pathways, or be transported to the nucleus of target cells to function as a DNA-binding factor. After IL-33 binds to its receptor complex, activated signals transmits into cells, and activates NF-κB and mitogen-activated protein kinase (MAPK) through a series of downstream signaling molecules such as IL-1-associated protein kinase, myeloid differentiation factor 88, and TNF receptor-associated factor 6 (153).

IL-33 has been reported to have neuroprotective effects through inhibiting inflammation *via* ST2 (a member of the IL-1 receptor family)/IL-33 signaling (154, 155). After ischemic brain injury, IL-33 expression in mature oligodendrocytes and astrocytes is increased. Interleukin-33 also protects against ischemic brain injury by regulating microglia and regulatory T cell activity (156). Serum IL-33 has been proved to be a novel predictive biomarker of hemorrhage transformation and outcome in acute ischemic stroke (157). The expression of ST2 on microglia/macrophages increases after MCAO. ST2 deficiency can exacerbate neurobehavioral disorders and brain damage *via* shifting microglial polarization toward M1. Some traditional oriental medicine, such as celastrol, ameliorate ischemic stroke injury through promoting IL-33/ST2 axis-mediated microglia/macrophage M2 polarization (158). IL-33 also protects ischemic stroke injury by regulating specific microglial activities (159). IL-10 is an essential protective factor for the neuroprotection of IL-33/ST2 signaling. In the ischemic brain, intracerebroventricular IL-33 can activate the downstream Foxp3 *via* ST2 receptor to increase Treg proportions, which produce amphiregulin to activate epidermal growth factor receptor located in neurons, leading to better outcomes (160). In addition, systemic administration of Th2 cells to promote cytokines IL-33 and IL-4 can reduce acute brain injury after CIRI (154). Astrocyte lipogenesis increases IL-33 production in the peri-infarct region, which promotes BBB repair in the subacute phase of cerebral ischemic injury and improves long-term functional recovery (161). The long-term protective role of IL-33 in ischemic stroke may be partly associated to its regulation of splenic T-cell immune responses *via* inhibiting Th1 response and promoting Treg response (162). Although IL-17 has these neuroprotective effects, mice treated with IL-33 showed an exacerbation of post-stroke pulmonary bacterial infection associated with greater functional impairment and mortality after 24 hours, suggesting exacerbation of systemic immunosuppression after ischemic stroke (163).

## 17 Other ILs in Ischemic Stroke

IL-5 and IL-9 are decreased in severe stroke patients acute ischemic stroke patients with poor outcome than mild stroke (164). It had been indicated that IL-5 and IL-7 may be predictors of edema and infarct volume (165). In experimental stroke, expressions of IL-9 and its upstream stimulating factors has been confirmed to be increased (166). Anti-IL-9-neutralizing antibody can ameliorate ischemic stroke injury partially by alleviating the destruction of the BBB *via* down-regulation of astrocyte-derived VEGF-A (167). In OGD, IL-9 has a destructive effect on the BBB, partly by decreasing eNOS production (168). IL-21 polymorphism is related to the increased susceptibility to ischemic stroke possibly by upregulating gene expression (169). IL-21R-deficient mice have reduced collateral vascular connections and increased brain infarct volume, suggesting that IL-21R regulates collateral vascular anatomy and innate neuroprotection. The neuroprotective effects of IL-21R are mediated through the JAK/STAT signaling pathway and upregulation of caspase 3 (170). IL-22 exerts a protective action through regulating the JAK2-STAT3 pathway to improve oxidative stress, inflammation, and neuronal apoptosis following CIRI (171). IL-32 silence protects PC12 cells against OGD/R-induced injury *via* activation of Nrf2/NF-κB pathway (172). Increased serum IL-34 levels may be a novel diagnostic and prognostic biomarker in patients with acute ischemic stroke (173). Increased serum IL-37 in ischemic stroke patients is correlated with stroke recurrence (174) and 3-month functional prognosis (175). However, another study has illustrated that IL-37 exert protective effects by modulating post-stroke inflammation in the brain and periphery (176). Large randomized controlled trials are needed to further verify the role of IL-37 in ischemic stroke. The lower changes in IL-38 serum level lead to a poorer prognosis, indicating that IL-38 serum changes might be a novel early predictor factor for ischemic stroke prognosis (177) (141).

## 18 Conclusions and Perspectives

**Figure 4:**
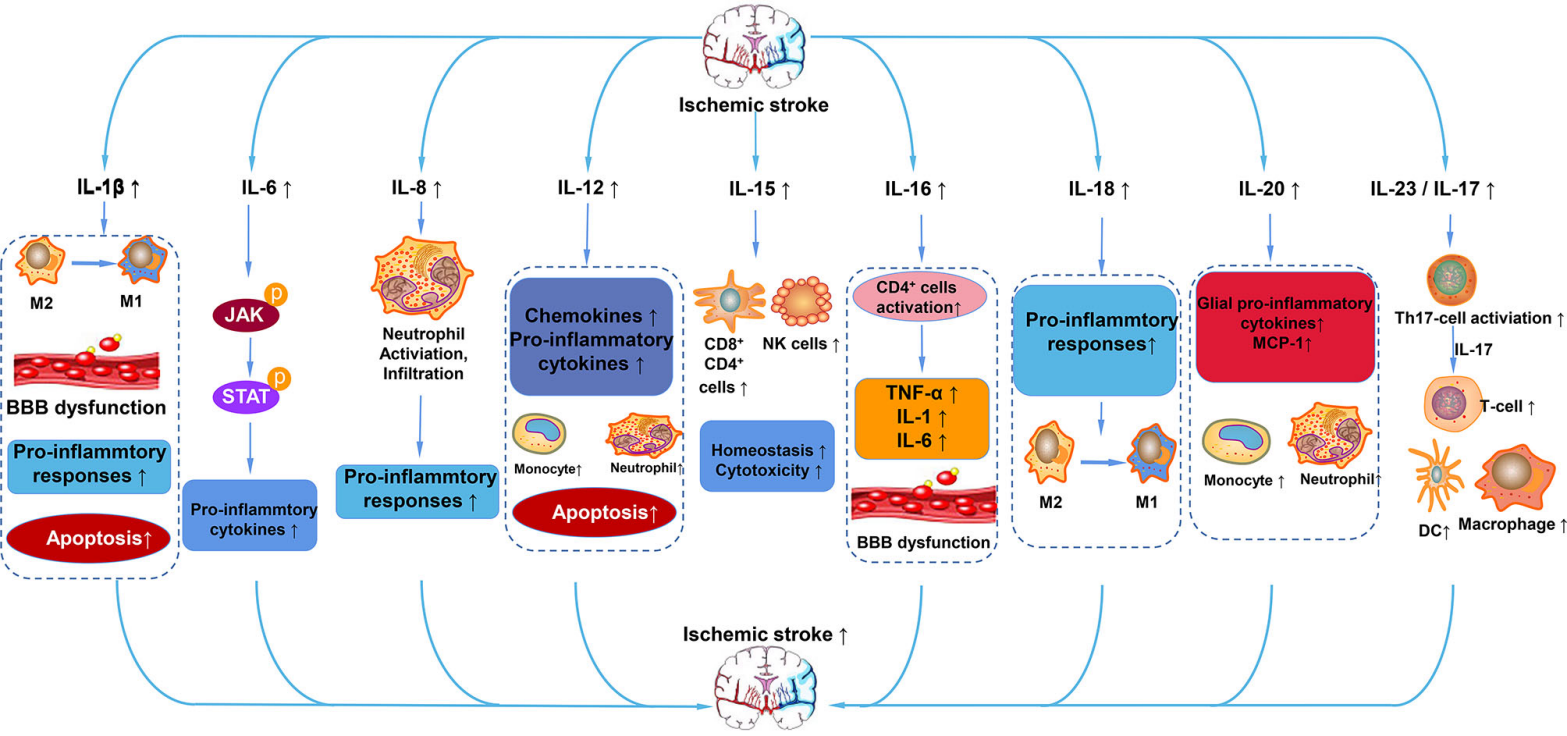
Pro-inflammatory ILs in ischemic stroke. After ischemic stroke, IL-1β aggravates cerebral infarction injury by polarization of microglia/macrophages to M1 phenotype, BBB dysfunction, and apoptosis. IL-6 activates the JAK/STAT pathway to promote the expression of pro-cytokines. IL-8 promotes the activation and infiltration of neutrophils into cerebral infarction. IL-12 promotes the expression of chemokines and pro-inflammatory mediators, promotes apoptosis, exerting a pro-inflammatory effect. IL-15 increases the number of CD4^+^, CD8^+^, and NK cells. IL-16 activates CD4^+^ cells and increases the levels of TNF-α, IL-1, and IL-6. IL-18 promotes the polarization of microglia/macrophages to the M1 phenotype and enhances the pro-inflammatory response. IL-20 promotes the expression of pro-inflammatory cytokines of MCP-1 and glia pro-inflammatory cytokines. IL-23/17 activates Th17 cells, which secrete IL-17 to increase the number of T cells, macrophages, and DC cells.

**Figure 5:**
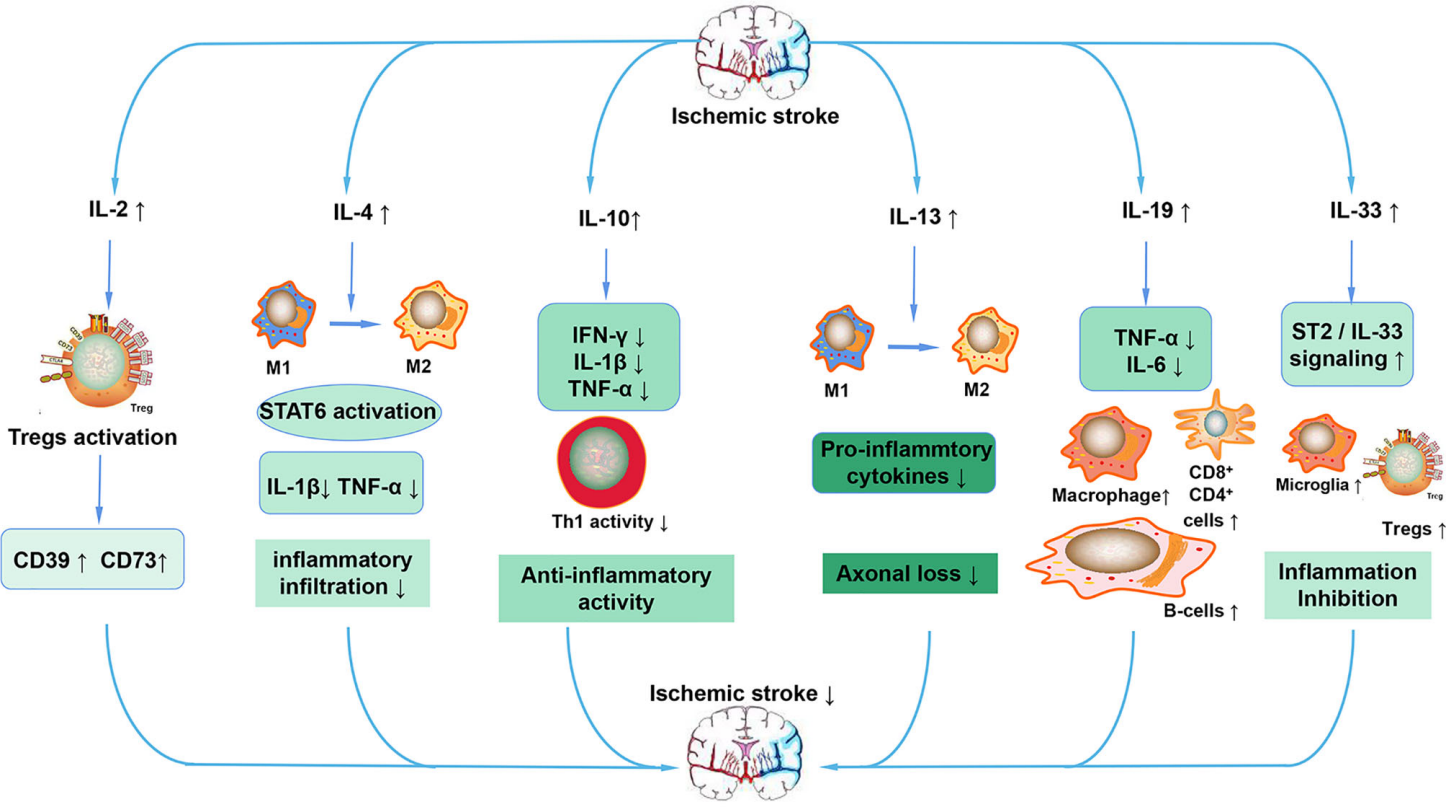
Anti-inflammatory ILs in ischemic stroke. After stroke, increased IL-2 activates Tregs, leading to the increased expression of CD39 and CD73. The increased IL-4 promotes the polarization of macrophages to the M2 phenotype and decreases the expression of pro-inflammatory cytokines such as IL-1β and TNF-α by activating STAT6. IL-10 also reduces the expression of pro-inflammatory cytokines, maintains the activity of Th1, ultimately exerting an anti-inflammatory action. IL-13 also increases the polarization of macrophages to the M2 phenotype, decreases the expression of pro-inflammatory cytokines and decreases the loss of neurons. IL-19 mainly preserves the reduced the number of CD4^+^, CD8^+^, and B cells and reduces the expression of pro-inflammatory cytokines. IL-33 inhibits inflammation after stroke mainly through the ST2/IL-33 signaling pathway.

In summary, we have briefly discussed the functional role of interleukins and their relationships with ischemic stroke. Based on the classification of the effect of interleukin on the immune response after stroke, interleukins can be roughly divided into anti-inflammatory and pro-inflammatory categories. IL-1β, IL-6, IL-8, IL-12, IL-15, IL-16, IL-20, IL-18, IL-23/IL-17 and so on play a pro-inflammatory role after ischemic stroke (**Figure 4**). ILs that have anti-inflammatory effects on ischemic stroke include IL-2, IL-4, IL-10, IL-13, IL-19, IL-33 and so on (**Figure 5**). However, the IL family contains so many ILs, the complicated roles of ILs in ischemic stroke cannot be discussed in detail in this brief overview. After ischemic stroke, ischemia leads to vascular endothelial damage and induces immune responses. The action of these ILs on local or systemic immune cells, or the interaction of these ILs, determines the progress of immune response in the ischemic brain. From a macroscopic perspective, it is their interactions that determine the degree of neurological impairment and clinical prognosis of ischemic stroke patients. Therefore, interleukins play an important role in the immune mechanism of ischemic stroke. This also urges us to make continuous progress and search in this field, in order to find a breakthrough method for clinical treatment of ischemic stroke, which is a worldwide problem, and bring hope to stroke patients.

## Author Contributions

HZ wrote the manuscript. JC, SQ, and SH designed this work. The figures were prepared by HZ. YL, YS, XH, and XX revised the manuscript. All authors contributed to the article and approved the submitted version.

## Funding

This study was supported by the National Natural Science Foundation of China (81870939 to XX).

## Conflict of Interest

The authors declare that the research was conducted in the absence of any commercial or financial relationships that could be construed as a potential conflict of interest.

## Publisher’s Note

All claims expressed in this article are solely those of the authors and do not necessarily represent those of their affiliated organizations, or those of the publisher, the editors and the reviewers. Any product that may be evaluated in this article, or claim that may be made by its manufacturer, is not guaranteed or endorsed by the publisher.
